# Tumor-Penetrating Peptides

**DOI:** 10.3389/fonc.2013.00216

**Published:** 2013-08-27

**Authors:** Tambet Teesalu, Kazuki N. Sugahara, Erkki Ruoslahti

**Affiliations:** ^1^Cancer Research Center, Sanford-Burnham Medical Research Institute, La Jolla, CA, USA; ^2^Laboratory of Cancer Biology, Centre of Excellence for Translational Medicine, Institute of Biomedicine and Translational Medicine, University of Tartu, Tartu, Estonia; ^3^Department of Surgery, College of Physicians and Surgeons, Columbia University, New York, NY, USA; ^4^Department of Cell, Molecular and Developmental Biology, University of California Santa Barbara, Santa Barbara, CA, USA

**Keywords:** synaphic targeting, homing peptide, tumor-penetrating peptide, neuropilin-1, αv integrins, C-end Rule

## Abstract

Tumor-homing peptides can be used to deliver drugs into tumors. Phage library screening in live mice has recently identified homing peptides that specifically recognize the endothelium of tumor vessels, extravasate, and penetrate deep into the extravascular tumor tissue. The prototypic peptide of this class, iRGD (CRGDKGPDC), contains the integrin-binding RGD motif. RGD mediates tumor-homing through binding to αv integrins, which are selectively expressed on various cells in tumors, including tumor endothelial cells. The tumor-penetrating properties of iRGD are mediated by a second sequence motif, R/KXXR/K. This C-end Rule (or CendR) motif is active only when the second basic residue is exposed at the C-terminus of the peptide. Proteolytic processing of iRGD in tumors activates the cryptic CendR motif, which then binds to neuropilin-1 activating an endocytic bulk transport pathway through tumor tissue. Phage screening has also yielded tumor-penetrating peptides that function like iRGD in activating the CendR pathway, but bind to a different primary receptor. Moreover, novel tumor-homing peptides can be constructed from tumor-homing motifs, CendR elements and protease cleavage sites. Pathologies other than tumors can be targeted with tissue-penetrating peptides, and the primary receptor can also be a vascular “zip code” of a normal tissue. The CendR technology provides a solution to a major problem in tumor therapy, poor penetration of drugs into tumors. The tumor-penetrating peptides are capable of taking a payload deep into tumor tissue in mice, and they also penetrate into human tumors *ex vivo*. Targeting with these peptides specifically increases the accumulation in tumors of a variety of drugs and contrast agents, such as doxorubicin, antibodies, and nanoparticle-based compounds. Remarkably the drug to be targeted does not have to be coupled to the peptide; the bulk transport system activated by the peptide sweeps along any compound that is present in the blood.

## Introduction

A major problem in systemic therapy is that only a small proportion of administered drug reaches its intended target site(s). Selective delivery of the drug to the target tissue can alleviate this problem. Affinity-based physical targeting (synaphic, pathotrophic, or active targeting) makes use of molecular markers that are specifically expressed at the target, and not elsewhere in the body, to accomplish selective targeting of systemically administered drugs (1). The desired outcome of the synaphic targeting is similar to topical application: increased local accumulation and lower systemic concentration of the therapeutic payload.

Synaphic targeting efforts have led to improved cancer drug delivery, but this approach only partially solves the selective delivery problem. Delivering a payload to a molecule specifically expressed on the surface of vascular cells in the target tissue can be effective because the vasculature is readily available for blood-borne probes. Thus, anti-angiogenic and vascular disrupting compounds can benefit from this approach. In fact, many of these compounds inherently target the vascular endothelium. An obvious example is antibodies that block the vascular endothelial growth factor receptors [VEGF-Rs, (2)]. These receptors are generally expressed at elevated levels in tumor vasculature. Hence the antibody (or other VEGFR ligand) has more binding sites in tumor vessels than elsewhere and could selectively carry a payload there. Less well known is that many of the natural and designed anti-angiogenic proteins highjack integrin-binding plasma proteins (fibronectin, vitronectin, fibrinogen) to selectively target the angiogenic tumor vessels. The anti-angiogenic proteins for which this has been shown include angiostatin, endostatin, anginex, and anastellin (3). However, besides tumor vessels, it is desirable to also target the tumor cells (and stromal cells) within the tumor. While delivering a drug to tumor vessels can improve the efficacy of the drug, the drug still has to extravasate and penetrate into the extravascular tumor tissue to reach the tumor cells. The technology we review in this article provides a solution to the tumor penetration problem. It can also help to deal with another, less appreciated problem of synaphic delivery: that the number of available receptors in a tumor is likely to be too low for the delivery of sufficient quantities of a payload drug.

## Vascular ZIP Codes in Drug Delivery

The endothelia of vessels in different organs, even when morphologically indistinguishable, are molecularly (and as a result, likely functionally) different [“vascular zip codes,” (4)]. Moreover, specific response patterns are activated in vascular cells during processes such as tumor growth, inflammation, tissue repair, and atherosclerosis. Many of the zip codes elicited by these processes are secondary to angiogenesis, the sprouting of new blood vessels from existing vessels. A common denominator is endothelial cell (and pericyte) activation, but each condition can also put an individual signature of the vasculature. One set of activation-related cell surface molecules, comprised of P-selectin, E-selectin, ICAM-1, and VCAM-l, is turned on by inflammation in venular endothelial cells and mediates leukocyte rolling and adhesion/emigration in response to inflammation (5, 6). Another signature set of cell surface molecules, comprising certain integrins, growth factor receptors, extracellular proteases, and extracellular matrix proteins, is expressed during angiogenesis, which is the main factor making tumor vasculature distinguishable from normal vasculature in the adult organism. Lymphangiogenesis and macrophage infiltration also contribute to tumor-related marker molecules (7).

*In vivo* phage display has been instrumental in establishing the extent of the molecular specialization in the vasculature and has contributed a number of new markers of tumor vasculature (4, 8). Bacteriophage can be genetically modified to incorporate random peptide sequences as fusions with the coat proteins at a diversity of about one billion variants per library, which is close to the total number of possible permutations of a random 7-amino acid sequence (1.28E9). For *in vivo* selection, a library of phage displaying random peptides is injected systemically into the animals, followed by removal of target organs, amplification of the bound phage, and subjecting the amplified pool to another round of selection. *In vivo* peptide phage screening combines subtractive elements (removal of phage displaying pan-specific peptides) with positive selection at the target tissue (9). This technology has yielded peptides with unique tumor-penetrating properties as discussed below.

## Tumor-Penetrating Peptides

### Modular structure of tumor-penetrating peptides

About 10 years ago, our laboratory identified a peptide, LyP-1 (CGNKRTRGC), with the ability to take the phage expressing it to the lymphatic vessels and hypoxic areas in tumors (10, 11). Surprisingly, the LyP-1 phage reached its targets in tumors within minutes of intravenous injection. Given that the phage is a nanoparticle and consequently diffuses slowly, diffusion did not seem to account for the rapid spreading within the tumor. It took the discovery of the CendR system, and the realization that it was responsible for the spreading within tumors of a more recently identified tumor-homing peptide, iRGD, to understand how these peptides penetrate into tumors (12, 13).

Tumor-penetrating peptides like iRGD and LyP-1 contain three independent modules: a vascular homing motif, an R/KXXR/K tissue penetration motif, and a protease recognition site. These modules cooperate to ensure a multistep, highly specific process of tumor-homing and penetration. The sequence of the prototypic tumor-penetrating peptide, iRGD, is CRGDR/KGPDC. We mostly use the K-variant, CRGDKGPDC, because it appears to provide stronger tumor-homing than the R-variant. Following systemic administration, the iRGD peptide is first recruited through its RGD motif to αv integrins, which are overexpressed on tumor endothelial cells. After the initial binding, proteolytic processing exposes the internal R/KXXR/K motif at the C-terminus of the truncated peptide. We have termed the R/KXXR/K motif the C-end Rule or CendR motif (pronounced sender) because of the requirement of C-terminal exposure for activity. The C-terminal CendR motif interacts with neuropilin-1 (NRP-1), and the NRP-1 interaction triggers the activation of a transport pathway (CendR pathway) through the vascular wall and through extravascular tumor tissue (12, 13). These peptides can take along both conjugated and co-administered payloads into the tumor parenchyma.

We came across the CendR phenomenon while screening phage libraries for peptides that would bind to and internalize into cells isolated from tumors grown in mice. We were initially disappointed to find that, independent of the starting library configuration (we used cysteine-flanked cyclic and linear random heptapeptide libraries), the selected peptides all looked similar; they all had a C-terminal arginine or lysine residues with another basic amino acid at the −3 position. However, we soon realized that the consensus motif, R/KXXR/K, had to be some kind of a master cell internalization signal and set out to study it. It is worth noting that, while our laboratory used the filamentous phage display system introduced by Smith (14, 15) in our early studies (8, 16), we later switched to the T7 phage. The important distinction is that in T7, the exogenous peptide is expressed at the C-terminus of the phage coat protein, whereas it is at the N-terminal end in the filamentous phage. Thus, the C-terminal truncations producing the CendR motif could only be selected for in the T7 system.

The binding and internalization of R/KXXR/K-displaying phage or synthetic nanoparticles required the presence of free C-terminal arginine or lysine residues as addition of additional amino acid residues to the motif or amidation of the carboxyl terminus resulted in loss of activity (12). In addition to the prostate cancer cell lines, the active CendR motif triggered binding, and internalization in many cultured tumor cell lines and in cells in suspensions prepared from normal mouse tissues. Studies on the prototypic active CendR peptide, RPARPAR, showed that the binding only takes place for the peptide made of L-amino acids and that the binding can be inhibited by excess of free peptide, suggesting the existence of a saturable receptor with a chiral recognition specificity. In contrast, cell-penetrating peptides, widely used for intracellular delivery of payloads *in vitro* are independent of position and chirality, and no specific receptors for them have been identified.

Affinity chromatography with RPARPAR identified NRP-1 as the main binding molecule for RPARPAR. NRP-1 is a trans-membrane receptor with major roles in cell migration and endothelial cell sprouting in blood vessels, while NRP-2 with a similar, but not identical binding specificity is abundant and plays an important role in lymphatic vessels (17, 18). NRP-1 is best known for its role as a co-receptor for certain members of the vascular endothelial growth factor (VEGF) and semaphorin families (19, 20). The NRP-1-binding VEGFs and semaphorins, and TGFβ, all have C-terminal CendR motifs. Tuftsin is an immonomodulatory peptide that has been shown to bind to NRP-1 [it has a C-terminal arginine residue, but lacks the complete CendR motif; (21)]. It induces vascular permeability (22), but no evidence on tissue penetration has been presented.

**Figure 1:**
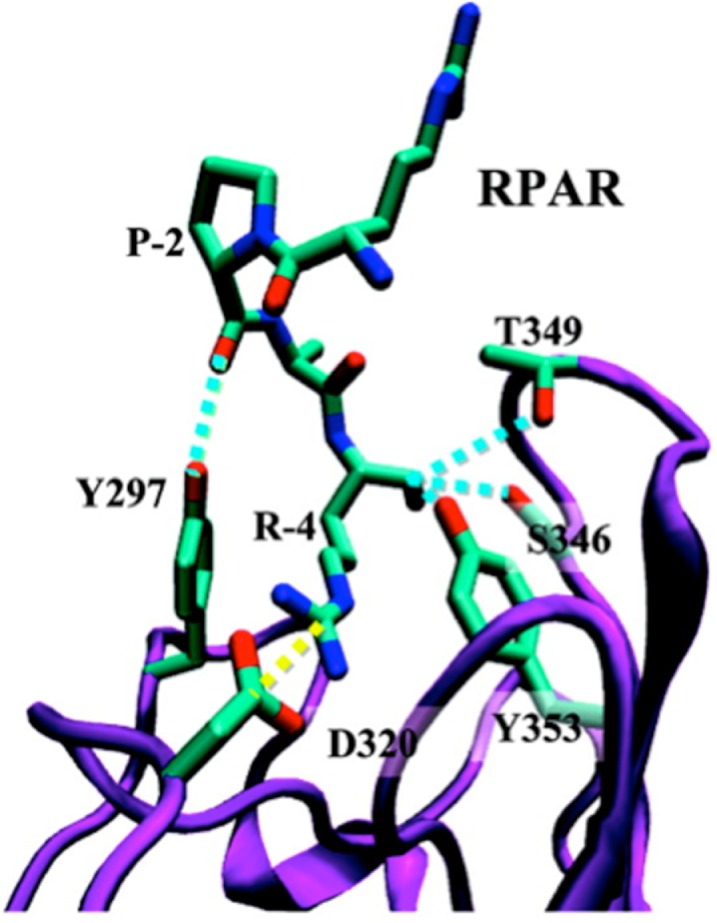
**Ribbon representation of the NRP-1-RPAR complex showing the most notable interactions found between the peptide and the binding pocket of NRP-1**. The ligand and the interacting side chains of the receptor are depicted as solid lines. NRP-1 backbone is shown in purple and RPAR backbone in green Hydrogen atoms are omitted for clarity. Specific interactions are drawn: hydrogen bonds are shown as blue discontinuous lines while salt bridges are marked by yellow discontinuous lines. Reprinted with permission from Haspel et al. (24). Copyright 2011 American Chemical Society.

The b1b2 domain of NRP-1 that contains the binding pocket for the CendR motif has been crystallized together with tuftsin (23). Molecular modeling studies show that peptides with a C-terminal CendR motif, such as RPARPAR fit well to the binding pocket, but do not provide an explanation for the role of the penultimate arginine residue, which remains outside the binding pocket [Figure 1; (24, 25)]. Perhaps this arginine could be engaging an as-yet unknown molecule in a three-way interaction with NRP-1.

Based on molecular simulations and phage binding to purified NRP-1 protein it appears that formation of a stable complex between a CendR peptide and NRP-1 requires interaction of the -2 and -3 residues with loop III of the b1 domain of the NRP-1, as in the case of RPAR, RRAR, RDAR, RPDR, RPRR, and RPPR (25). For a stable interaction to occur, loop III must be engaged in a pairwise interaction that stabilizes the interaction of the C-terminal carboxylic group with the CendR binding pocket in the b1 domain of NRP-1.

Interestingly, the D-conformer of RPARPAR is a poor fit with the binding pocket, suggesting that the D-Tat, even with a C-terminal arginine would not bind to NRP-1. The modeling studies also indicate that under some circumstances a cyclic peptide could fit into the binding pocket (24). Indeed, peptides built on a thermostable, protease-resistant cyclotide kalata B1 scaffold have been described that are thought to interact with NRP-1 as intact cyclic peptides (26). These modeling studies provide a basis for *in silico* screening of CendR analogs and evaluation of low molecular weight compounds resulting from high throughput screening. The molecules that bind to the CendR binding pocket on b1b2 domain of NRP-1 will be either acting as agonists or antagonists with potential applications in cancer drug delivery, and in diseases associated with elevated vascular permeability and pathogen spreading in tissues (see below).

A wide range of other receptors have been reported to use NRP-1 as a co-receptor, earning NRP-1 designation as a “hub” receptor (27), but it is not clear whether the ligands of these receptors use the CendR motif binding site for docking to NRP-1. Whereas NRP-1 can signal independently of other signal-transducing receptors, the primary role of NRP-1 is believed to be acting as a co-receptor that ensures the recruitment and presentation of various ligands to the effector receptors. NRP-1 is overexpressed in many cancer cell lines, where it is implicated in migration, proliferation, and survival. NRP-1 is overexpressed in tumors, both in cancer cells and in stromal cells, and is implicated in development and maintenance of the tumor vessels and in tumor growth and progression (28, 29). NRP-1 is a target of anti-cancer therapy with antibodies and peptide-bound therapeutic agents (30–34). However, as the NRPs are also widely expressed in normal vessels, the overexpression in tumors will only afford a degree of tumor specificity. Another aspect is that in bloodstream, plasma proteases carboxypeptidases [e.g., carboxypeptidase M and N; (35)] rapidly remove C-terminal arginine residues, thus limiting the efficacy of systemic active CendR peptides in tumor drug delivery. In contrast, the localized tumor-specific proteolytic activation of the cryptic CendR motif of our tumor-penetrating peptides results in tumor-specific activation of a cell and tissue penetration pathway.

### The CendR pathway

The ability of VEGF and semaphorins to increase vascular permeability has been recognized for some time. Dvorak and Feng (36) showed that VEGF induces the formation of a network of tubular vesicles in endothelial cells they named the “Vesiculo-vacuolar organelle,” and presented morphological evidence that these interconnected vesicles could form a pathway though cells. The complicating factor in interpreting these results is the activity of the main signaling receptors for VEGF (VEGF-Rs) and for the semaphorins (plexins). CendR peptides allow one to study the NRP binding in isolation of other receptors and have made it possible to show that NRP-1 [and NRP-2, (37)] activate a trans-tissue transport pathway.

The uptake of the payload of CendR peptides into intracellular vesicles shows that the entry into cells is through an endosomal route. Moreover, the rapid penetration of the payloads of tumor-homing CendR peptides into tumors *in vivo* and *ex vivo*, and its energy dependence (13, 37, 38) shows that this is an active transport pathway, not one dependent on diffusion. The CendR pathway may be distinct from the known endosomal pathways, but at this point the evidence to that effect is limited to the use of various pharmaceutical inhibitors of the known pathways (12). The extravasation and tumor-penetration activities of iRGD suggest that the payload of the CendR endocytic vesicles is also at least partially released from cells by fusion of the endosomes with the plasma membrane. We have not yet observed the exocytosis phase of this presumed transcellular pathway, but the rapid tissue penetration of the CendR payloads support of this possibility. However, we cannot exclude that an alternative pathway such as propelling cell surface-bound payload forward by the cell membrane or membrane projections. Genetic and proteomics studies are underway to elucidate the cellular molecular basis of the CendR pathway.

Our discovery of the CendR tissue transport pathway raises the fascinating question of the physiological function of this pathway. While the focus so far has been on how this pathway might be used in drug delivery, it obviously does not exist for this purpose. One possibility is that it facilitates the transfer of nutrients to cells that are far from blood vessels or otherwise under duress. The overexpression of NRP-1 in tumors suggests that supplying nutrient deficient/hypoxic areas in tumors may be yet another way tumors make use of a physiological pathway to foster their own growth. The CendR pathway may have been hijacked by viruses and microbial toxins for cell entry and tissue spreading. Cleavage of a viral surface proteins and pro-toxins by host proteases (most commonly furins and related enzymes) at sites that create an active CendR motif is a recurrent theme seen in many pathogens. Examples include the Human T-lymphotropic virus-2, Crimean–Congo hemorrhagic fever virus, tick-born encephalitis virus, and Ebola viruses, as well as anthrax toxin (39–43). CendR sequences are also present in snake and bee venoms (e.g., mellitin), and may contribute to the spreading of these toxins in tissues.

Vascular edema is associated with many diseases (hemorrhagic virus infections, sepsis, and vascular leak syndromes). Several proinflammatory vasoactive (poly)peptides capable of increasing vascular permeability display a functionally important arginine residue at their C-terminus. Examples include complement C3a and C5a anaphylatoxins (C-terminal sequences ASHLGLAR and KDMQLGR, respectively) as well as kinins (bradykinin and kallidin, which have an identical C-terminal sequence, RPPGFSPFR). Intriguingly, we have observed that phage that display peptides corresponding to the C-terminal amino acids of C5/3a and bradykinin bind to the recombinant b1b2 domain of NRP (in preparation) and that the binding is reversed by an excess of the free peptide. It remains to be seen whether the NRP/CendR axis plays a role in the activity of C3/5a, bradykinin, and/or other vasoactive peptides.

### Designer peptides for CendR pathway activation

Having worked out the two-motif requirement for a tumor-homing peptide to have CendR activity, we tested the universality of the concept by designing a new peptide with such activities. We used as the starting point the NGR tumor-homing motif previously identified by our laboratory (44, 45), which recognizes a form of aminopeptidase N in angiogenic tumor vessels (46, 47). We added a second arginine to the NGR motif to convert it into the CendR motif, RNGR and embedded that motif in the iRGD framework by replacing RGDK with RNGR. The resulting peptide, iNGR (CRNGRGPDC) has all the properties of iRGD, except that its tumor recruitment is not mediated by integrin but another receptor, presumably aminopeptidase N (48). We have also designed tumor-homing CendR peptides by arranging in tandem a CendR motif, a proteolytic cleavage site for a tumor-associated protease that cleaves after a basic residue, and a tumor-homing motif (Teesalu et al., in preparation). These peptides also home to and penetrate into tumors. A construct created to deliver a non-specific cell-penetrating peptide, appears to serendipitously follow this design (49). Whether these tandem tumor-penetrating peptides are as effective as the peptides in which the homing motif and CendR motif overlap remains to be seen. The iRGD and LyP-1 peptides lose their affinity for the primary receptor [αv integrins for iRGD and a mitochondrial/cell surface protein p32 for LyP-1 (7) after the proteolytic cleavage that activates the CendR motif has taken place (13, 37)]. The resulting release of the peptide from the primary receptor may facilitate subsequent binding to NRP-1 and make the primary receptor available for binding of another intact peptide. Peptides with tandem motifs would lack this latter feature. Another possible design for CendR activation would be blocking the C-terminus with a chemical group other than an amino acid or peptide. One can envision peptides, the CendR activity of which is triggered by a phosphatase, demethylase, sulfatase, etc. To the extent such an enzyme is specific for the target tissue, new useful probes could be created.

## Drug Delivery with Tumor-Penetrating Peptides

### The drug penetration problem

To reach tumor cells and tumor-associated parenchymal cells (e.g., tumor-associated fibroblasts, macrophages), drugs must cross the vascular barrier and penetrate into the extravascular stroma. Cancerous tissue is heterogeneous, with striking regional differences in tumor structure (leaky vasculature and defective lymphatics, which causes buildup of interstitial fluid pressure in the tumor), and physiology (e.g., inflammation, fibrosis, hypoxia, acidity). These features translate into steep drug gradients and variability in the uptake and distribution of anti-cancer drugs within tumor parenchyma (50). For example, evaluation of doxorubicin distribution in tumors after systemic administration showed that the concentration of this drug decreases exponentially with distance from tumor blood vessels, reaching half of its perivascular concentration at a distance of about 40 μm (51). The distribution of trastuzumab (Herceptin) in breast tumor xenografts is also highly heterogeneous with many tumor cells exposed to no detectable drug (52). To some extent, the tumor drug delivery challenges are alleviated by the Enhanced Permeability and Retention (EPR) effect – accumulation of compounds (typically liposomes, nanoparticles, and macromolecular drugs) in tumor tissue more than they do in normal tissues. The underlying causes of the EPR effect are abnormal structure and function of tumor vessels: poorly aligned endothelial cells with fenestrations, deficient pericyte coverage, and lack of lymphatic drainage. However, EPR is highly variable as it is influenced by differences between tumor types and heterogeneity within individual tumor. Tumor interstitial pressure (IFP) depends on integrity of blood and lymphatic vessels, tumor cell proliferation, deposition of matrix molecules, and interaction of cells with the matrix molecules. The difference between tumor microvascular fluid pressure and IFP determines intratumoral convection fluxes that have a major influence on the vascular exit of the compounds over 10 kDa. Intratumoral fluid pressure gradients can be in some cases favorably influenced by vasodilatory compounds such as bradykinin, endothelin, and calcium channel antagonists, to allow better tumor perfusion and increased drug delivery (53). Other approaches include dissolving extracellular matrix with enzymes such as collagenase or hyaluronidase (54), or killing or inhibiting the activity of tumor-associated fibroblasts (55). Obviously, the delivery of enzymes and drugs aimed at lowering the IFP to the tumor parenchyma faces the similar tumor penetration challenges seen for the cancer drugs.

### CendR-enhanced drug delivery

The tumor-homing CendR peptides provide a solution to the drug penetration problem. A probe or drug attached to iRGD or LyP-1 is delivered to extracellular tumor tissue more effectively than the drug alone. We have extensively demonstrated the tumor penetration with fluorescein (FAM)-labeled peptides. Intravenously injected FAM-iRGD, LyP-1, and iNGR are found dispersed in tumor parenchyma minutes after administered, whereas FAM-labeled inactive control peptides do not appear in the tumors at all. FAM-labeled homing peptides that lack a CendR motif bind to the blood vessels, but do not penetrate into the rest of the tumor (10, 11, 13, 48). Remarkably, iRGD and LyP-1 have quite different distributions within tumors, presumably reflecting the expression of their primary receptors in different tumor compartments (7, 10, 13). The effect of the cryptic CendR motif is vividly illustrated by the differences between iRGD and conventional RGD peptides, such as CRGDC and cycloRGDfK. While iRGD payload, even a poorly diffusing nanoparticle, readily enters tumor parenchyma, the conventional RGD peptides only take their payload to the tumor vessels (13, 38). LyP-1 and CGKRK, a peptide we have recently shown to also use p32 as its receptor but lack the CendR activity (56) show a similar difference (11, 57).

**Figure 2:**
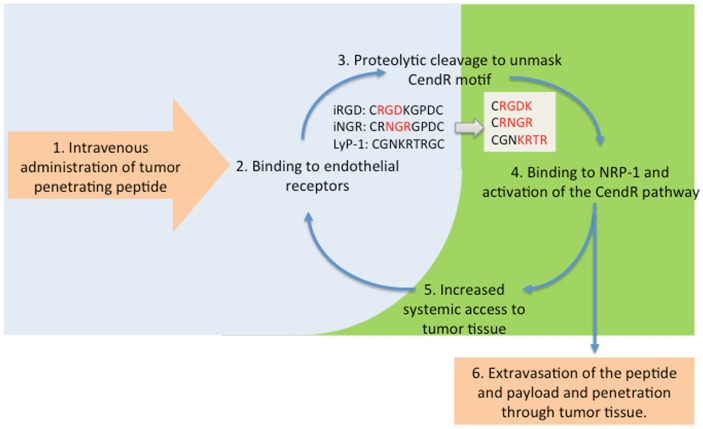
**The tumor penetration cycle of CendR peptides**. Following systemic administration, tumor-penetrating peptides are initially recruited to tumor blood vessels (2) followed by proteolytic processing to unmask the CendR motif, and activation of NRP-1-binding (3, 4). NRP-1 engagement triggers extravasation of the processed peptide and payload and triggers a bulk transport process that increases delivery of payloads (6) and systemic accessibility of blood-borne compounds, including unprocessesed tumor-penetrating peptides for progressive penetration into tumor tissue (5).

The observations with the fluorescent probe described above prompted us to study the ability of iRGD and the other CendR peptides to enhance the delivery of actual anti-cancer drugs to tumors. We have shown that therapeutics as diverse as a small molecular weight drug (doxorubicin), trastuzumab (anti-Her2 antibody), and the nanoparticle drugs Abraxane and Doxil can benefit from iRGD-enhanced delivery (13, 38). In showing this, we mostly made use of a unique property of iRGD and other similar peptides; they can enhance tumor penetration of payloads that are not attached to the peptide, just administered at the same time. The reason is that iRGD activates a bulk transport pathway that moves along any compound present in the blood when the system is active. The scheme in Figure 2 illustrates this principle.

Timing measurements have shown that the CendR pathway is active for about 1 h, with peak activity about 30 min after the administration of the peptide (38). The timing agrees with the half-life of the peptide in the blood, which for a peptide of this size can be expected to be about 10 min (58). The main reason for the short half-life is elimination of the peptide through filtration into the urine. It remains to be determined whether prolonging the half-life of the peptide would further enhance drug delivery into tumors. We compared the efficacy of directly conjugating the drug to iRGD and the co-administration with Abraxane as the drug. Both methods gave significantly higher anti-tumor activity than the drug alone, and seemed equally effective in this regard in the tumor system we studied (38). However, it should be noted that the number of receptors at the target limits the efficacy of the conjugated delivery. Calculations show that a gram of tumor tissue is not likely to have more than a few picomoles of any given receptor available for targeting of drugs with probes coupled to the drug (1). Most drugs to be effective require greater concentrations than could be delivered to this small an amount of receptor. The co-administration mode does not have this limitation, as only the triggering of the trans-tissue transport pathway is needed. Another major advantage is that it is not necessary to conjugate the drug to the homing peptide, which would create a new chemical entity with the attendant regulatory hurdles.

LyP-1 coupled to Abraxane nanoparticles also increased the efficacy of the drug (59) and iNGR promoted the activity of doxorubicin in a mouse tumor model in a way similar to iRGD (48), by a factor of about 3. Importantly, the iRGD work with doxorubicin showed that there was no change in the main side effect of this drug, cardiotoxicity. This side effect was nearly eliminated by a threefold reduction of the drug dose. Thus, the tumor-penetrating peptides can be used both to enhance the activity of anti-cancer drugs, or lowering the side effect with the same anti-cancer activity, or some of both.

The tumor-penetrating peptides can also enhance tumor imaging, as demonstrated by coating iron oxide nanoparticles with iRGD for MRI imaging. iRGD gave stronger images than a conventional RGD peptide, CRGDC; the main difference was that iRGD spread into the whole tumor, whereas only highlighted the tumor vessels (13). LyP-1 has been used in optical imaging of tumors (11, 61) and atherosclerotic plaques (60), as well as in MRI and PET imaging of plaques (61). LyP-1 homes to and penetrates into activated macrophages in tumors and atherosclerotic plaques (60, 61) revealing a similarity between the macrophages in tumors and the plaques (61). LyP-1 has also been shown to selectively accumulate in tumor-draining lymph nodes prior to the arrival of tumor cells, defining a premalignant niche in tumors (62).

## Conclusion and Future Prospects

The discovery of tumor-penetrating peptides has led to the identification of a new trans-tissue transport pathway, the C-end Rule or CendR pathway. The physiological function of the CendR pathway and its molecular workings are obviously important questions to be answered in future studies. Activating the pathway in a tumor-specific manner, which is accomplished with peptides the CendR motif of which is activated in tumors, provides a way of increasing the activity of anti-cancer drugs and enhancing tumor imaging. Thus, the tumor-penetrating CendR peptides represent a potentially significant advance in cancer treatment.

## Conflict of Interest Statement

Tambet Teesalu, Kazuki N. Sugahara, and Erkki Ruoslahti are shareholders in CendR Therapeutics Inc., and Erkki Ruoslahti is a shareholder in EnduRx Pharmaceuticals. The companies have rights to some of the technology described in the paper.
